# Authentic teaching and learning through synthetic biology

**DOI:** 10.1186/1754-1611-1-8

**Published:** 2007-12-27

**Authors:** Natalie Kuldell

**Affiliations:** 1 MIT, Department of Biological Engineering, 77 Mass Ave, 16-325, Cambridge, MA 02139, USA

## Abstract

Synthetic biology is an emerging engineering discipline that, if successful, will allow well-characterized biological components to be predictably and reliably built into robust organisms that achieve specific functions. Fledgling efforts to design and implement a synthetic biology curriculum for undergraduate students have shown that the co-development of this emerging discipline and its future practitioners does not undermine learning. Rather it can serve as the lynchpin of a synthetic biology curriculum. Here I describe educational goals uniquely served by synthetic biology teaching, detail ongoing curricula development efforts at MIT, and specify particular aspects of the emerging field that must develop rapidly in order to best train the next generation of synthetic biologists.

## Review

### Teaching opportunities and challenges specific to synthetic biology

"Plant a carrot get a carrot, not a Brussels sprout" sings a musical theater character in *The Fantasticks *[1], aptly contrasting the predictability of gardening over childrearing. Map this idea to education and it seems teaching more closely resembles horticulture than parenting. Traditional metrics and standards around education often restrict educators to fixed lesson plans and syllabi, many of which have not changed since teachers were students themselves. Such preset teaching agendas enable students to achieve predictable, measurable learning outcomes and provide a framework to till a uniform garden of carrots (or geneticists or physicists or computer programmers). This educational framework, however, leaves little to no room for students to wrestle with the flexible thinking and uncertainty that characterize true discovery. It minimally connects information at the boundaries of traditional disciplines. An alternative teaching model establishes collaboration between teacher and student, providing a more student-centered learning experience than traditional didactic or Socratic methods. Though the measurement tools for this kind of collaborative learning are blunt, it remains clear that an effective and lasting education must inspire student innovation, creativity, and confidence giving rise to a garden full of individuals who are independent, skillful and responsible thinkers.

Synthetic biology is particularly well suited to collaborative and integrated learning but it should not be automatically lumped with all "interdisciplinary" approaches to problem solving. The catch-phrase "interdisciplinary" has grown popular in both education and research [2-7]. Reductionist approaches to understanding that tease systems apart are currently less fashionable than integrative efforts that draw from traditionally distinct specialties to more fully describe the whole. However, despite seeming inherently interdisciplinary, synthetic biology is, in fact, not. It does not simply put biologists and engineers in adjoining offices and wait to see what fireworks erupt at the water cooler. Instead, synthetic biology is a distinct discipline that requires its practitioners to work in ways remarkably different from the work that defines any traditional niche. Biologists who come to synthetic biology must manage complexity, rather than describe and celebrate it. Engineers must build using material under evolutionary pressures. Students who enter synthetic biology perceive the promise and limitations of the emerging discipline and because they have yet to categorize themselves as either "engineer" or "scientist," these students do not see the need to collaborate as much as they see the need to parse out the problems themselves and then systematically develop the skills to solve them.

An equally relevant pillar of synthetic biology education is its demand for awareness of real world dynamics. There is already good evidence that emotional, political and economic pressures as well as technical achievements will guide the development of synthetic biology [8-10]. As students become active members of the synthetic biology community they will be navigating both inside and outside the Ivory Towers. Consequently they will need an awareness of the public mindset, articulate answers to questions of misapplication and mistakes, and a persuasive approach to marshal support for their inventions. Vocabulary and techniques for social engineering can be taught as extensions of current persuasive writing and public speaking initiatives, and as with the synthetic biology efforts described below, integrated into problem-based learning frameworks. The stakes and rhetoric around synthetic biology are high, and educational efforts that fail to equip students for this aspect of the emerging discipline are unsound.

The newness of synthetic biology makes "typical" instruction nearly impossible. For example, how can a teacher properly assess "mastery of subject matter" when the foundational framework and professional competencies of the field have yet to be determined? Effective communication skills and sophisticated reasoning may distinguish experts from novices [11] and so might be considered appropriate readouts for accomplishment, but the measures for success in these areas are imprecise and difficult to apply [12]. Nevertheless, several programmatic educational efforts are underway that powerfully illuminate the promise of synthetic biology. All are simultaneously hampered and energized by the newness of the field. All aspire to teach great literature while the state of the art is a few rhymed couplets [13].

### Synthetic Biology 101

Audiences who have requested programmatic material for synthetic biology education include iGEM participants (see below), college and university biological engineers, grade school teachers, computer scientists, policy makers, and members of existing scientific and engineering communities. All have reasons and interest in making biology easier to engineer, but there can be no single template suitable for teaching such diverse audiences. Nevertheless, a core curriculum around synthetic biology can be described. It will include but not be limited to the following learning goals:

#### 1. Students will design biological systems in skillful and responsible ways

There is a lot embedded in this goal. Primarily it is intended to specify the engineering equivalent of scientific, hypothesis-driven research. It can be loosely translated as: why should I build it and how? Students must wisely choose the best technology to solve a given problem and should know that synthetic biology will not always be the answer.

#### 2. Students will design, specify and whenever possible implement their design

When they learn by building, students will pinpoint stumbling blocks to the predictable engineering of biology and some may take on the task of solving them. For example, computer-aided design of biology resembles that of the automobile industry decades ago when cars were slammed into walls to gather safety data about head-on collisions. Short of building a biological system, it is difficult to anticipate its performance.

#### 3. Students will conscientiously use materials

Knowing that the natural world can be intentionally changed in major ways, students must identify for themselves what is worth changing. The synthesis of destructive agents should never be the desired outcome.

#### 4. Students will define the values, culture, safety practices, and organizational community of the field

With synthetic biology still in its adolescence, community definition and building must be an explicit goal and students must feel empowered to meet it.

### Pilot and ongoing educational efforts

Practically speaking, how can these educational goals be met? There is no single source or textbook to describe synthetic biology; consequently, new learners rely on a variety of sources for their introductory and foundational information. Complete newcomers may find relevant information in websites [14-17], blogs [18,19], lay press articles [20,21] and meeting reports [22-25]. With some basic understanding of biology and engineering, learners can tap into the primary literature, including some of the seminal papers [26,27]. The initiated can also learn from Campbell and Heyer, who nobly include a chapter on synthetic biology in their college-level textbook [28]. Spanning the divide between novices and traditional students are outreach efforts such as the adventures of Systems Sally, Device Dude and Buddy in comics [29] and animations [30]. These communicate foundational ideas in accessible and entertaining formats.

For experiential learning, the summer-long International Genetically Engineered Machines (iGEM) competition is a model [31,32]. In it, synthetic biology is practiced by teams of students affiliated with colleges and universities around the world. The iGEM competition has grown rapidly, from 13 participating teams in 2004 to 55 teams representing more than a dozen countries in 2007. Genetic parts from the Registry of Standard Biological Parts are mailed to participating teams who then try to build novel genetically-encoded systems. Deeply discounted DNA synthesis is available to teams who desire parts not found at the Registry. In the spirit of open source biology, the iGEM coordinators have produced educational materials that are freely available (including podcasts and presentations from "teach the teachers" workshops) [33] and have further required that participating teams document their projects on a public wiki. Some remarkable achievements have been realized through the work of iGEM teams, including a bacteria-based photography system and bacteria that produce pleasantly fragrant compounds depending on the strain's growth state [34]. The former project was published in a peer-reviewed journal [35] and the iGEM students themselves presented the latter at the 2007 annual meeting of the Institute for Biological Engineering [36].

Several formal classes in synthetic biology are under various stages of development at colleges and universities around the US, including Brown, Davidson, Harvard, MIT and UC-Berkeley [37-41]. I team-teach a sophomore level laboratory subject at MIT called Laboratory Fundamentals of Biological Engineering [42]. Drew Endy and I draw teaching material for this course from ongoing efforts in synthetic biology. For example we teach a genome engineering unit drawn from T7.1, the successful redesign of the T7 bacteriophage genome. T7.1 is an example of genetic "refactoring," that is an engineered surrogate genome rewritten to maintain biological function while also being easier to study and extend [43]. Our students use a filamentous phage (M13) as genetic substrate to manipulate with traditional molecular tools. They also specify a "clean" genome free from overlapping reading frames that could be more useful for building batteries and electrochromic devices [44,45]. Finally, students consider modifications to the genome of the bacterial host ("chassis design"), evaluating performance of the natural and modified phage on hosts with natural and minimal genomes [46]. These lessons distinguish subtle and wholesale changes to nature and students struggle with the benefits and uncertainties of each approach. Intrinsic to this study are foundational engineering ideas such as modularity and standardization, and students describe their experimental work not in terms of data collected but rather as an example that moves the whole field of synthetic biology forward. Moreover, their work is "actionable" since the best designs for the bacteriophage genome are synthesized and then tested by the students in the lab within the timeframe of a one-semester class [47]. Finally, the curriculum inspires some robust discussions within the class and among the faculty around biosafety and security, for example what will happen when sophomore students a few years from now tackle gene therapy by refactoring a lentivirus rather than a bacteriophage?

**Figure 1:**
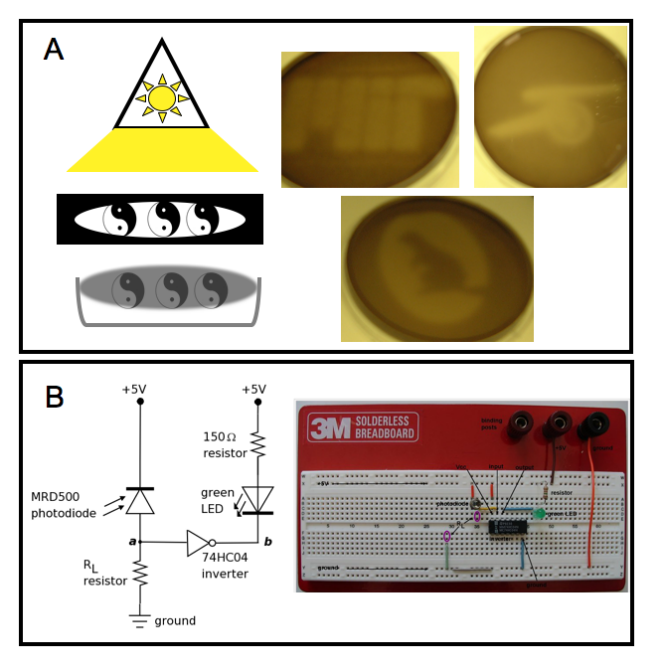
**Teaching Synthetic Biology**. The bacterial photography system (35) was used as the experimental overlay for an undergraduate curriculum in system engineering. (A) The experimental set-up (left) for the bacterial photography system includes a 660 nm light source that shines through a black and white mask onto a lawn of engineered bacteria. Overnight the bacteria precipitate a colored compound in the media depending on whether the cells are exposed to the light or hidden from it by the mask, giving rise to images like the student's examples that are shown (right). (B) Electronic components can be used to describe the genetic circuitry of the bacterial photography system (left). The light sensor function that is encoded by proteins in the bacterial cells is represented as a photodiode and inverter. An LED represents the actuator function. Students can vary the resistors on a breadboard (right) to consider design issues such signal matching and parts optimization.

Our course at MIT includes a second synthetic biology laboratory module based on the bacterial photography system in which cells serve as pixels, turning the media brown when grown under light of a particular wavelength [35,48] (Figure 1A). Students choose images to develop, optimize the camera set-up and modulate an experimental parameter to better understand the system. We've seen students choose images of the MIT beaver, of the Pittsburg Steeler's football logo, and of Al Pacino's Scarface poster. We've seen students make pinhole cameras from small cardboard boxes covered with tape and from Styrofoam ice chests with black paper interiors. Experimental parameters that the students have varied include strain growth conditions like cell density or temperature as well as modifications to the camera set up itself, for example growing their developing images a different distance from the light source or adding a reflective surface under their growing cells. Additionally, students demonstrate their understanding of the system's design by expressing the relevant genetic elements as electronic components, using a photodiode to represent the light sensing proteins in the biological circuit, and so on. By building and perturbing the circuit on a breadboard (Figure 1B), students can appreciate the divide between biology and engineering. For instance, it takes only seconds to replace one resistor with a different one, and the outcomes of such manipulations are often predictable since electronic components are well characterized and clearly documented. By contrast, to replace a "part" in a biological circuit requires days of planning, cloning, verifying and testing. When successful, though, the biological instance of the circuit is a self-replicating template that is far easier and cleaner to mass-produce than the electrical circuit.

The "ah-ha" factor associated with both the M13 redesign and the bacterial photography series of experiments is particularly gratifying to both students and instructors. The systems themselves are charismatic and relevant to future biological applications since light is minimally disruptive to most cells and phage is currently used in nanotechnology [49]. The student's experiments model the integrative nature of synthetic biology, with expressions of genetic logic, information processing, abstraction, and parts optimization. What they lack, however, is the predictability seen in exemplary curricula. We've seen many of the student's M13 redesigns fail to make functional phage. We've seen many of the student's bacterial photographs fail for reasons that cannot be explained. Additionally both the M13 redesign template and the bacterial photography system need standardization of their genetic parts to exemplify a true biological engineering effort. As a patch, we ask our students to directly address these limitations in their writing assignments, requiring they self-identify as synthetic biologists and present their viewpoints and solutions as stakeholders [50].

While to date neither of these experimental modules in synthetic biology is sufficient, we hope to integrate these fledgling units into a more complete educational effort. We expect to include an experimental module that refines parts, another that builds and measures genetic devices and a capstone module that explores application spaces for completed systems. We have, additionally, begun to develop a complementary "lab-free" curriculum to introduce biological engineering design [51] and some instructional guidelines that distinguish synthetic biology (Table 1).

**Table 1:** Lessons learned from teaching synthetic biology

**DO**	
	**encourage students to precisely and carefully articulate their views**
	**require students to self-identify as a member of the synthetic biology community**. As stakeholders, they can test their comfort with the advantages and risks, practice and evaluate the rhetoric around thorny issues.
	**raise the thorny issues**. Sweeping under the rug all questions of security, safety, dual-use, and ownership is a disservice to all. The current crop of students will almost certainly face a biological threat in their lifetimes, and we will be relying on them to react sensibly and constructively. Teachers may themselves have to move outside their comfort area of expertise to address such questions. This, too, is a good outcome.
**DON'T**	

	**conflate synthetic biology with existing disciplines **such as genetic engineering and nanotechnology. Without question, there is overlap, sometimes profound overlap, making clear definitions impossible, but to teach a "synthetic biology lab" which moves a luciferase gene into *E. coli *is incorrect. Teach PCR, teach bacterial transformation, but teach them as tools that are common and available, not as the core of synthetic biology.
	**shy away from criticizing the fledgling efforts that synthetic biology has itself celebrated. **The Registry is great but far from perfect. Ask students to make it better. The production of artemisinin has tremendous promise but is a hybrid effort that resembles but doesn't exemplify the engineering of biology. Confirm that students see the distinction and what they would implement as v2.0.
	**overstate what synthetic biology may do **or anticipate that every garage will be filled with designer biology. Nature is a harsh critic, so while the widespread availability of biological components will surely give rise to a cadre of volunteer debuggers and verifiers, it's reasonable to think that their failure rate will overwhelmingly exceed their successes, at least in the near term.

## Conclusion

Synthetic biology, with its inclusive content and uncertain outcome, can be used to educate the skilled and responsible thinkers we hope to produce. The newness of the terrain engages students as stakeholders who learn that their viewpoints matter and that their ideas are actionable. Teaching synthetic biology is hampered by the limited number of robust systems that can be converted to teaching materials and by the dearth of standardization and characterization in existing synthetic biology exemplars. Nonetheless, it's today's students who can contribute to the growth of the field and who will soon become practitioners poised to realize the positive outcomes for biology by design.
